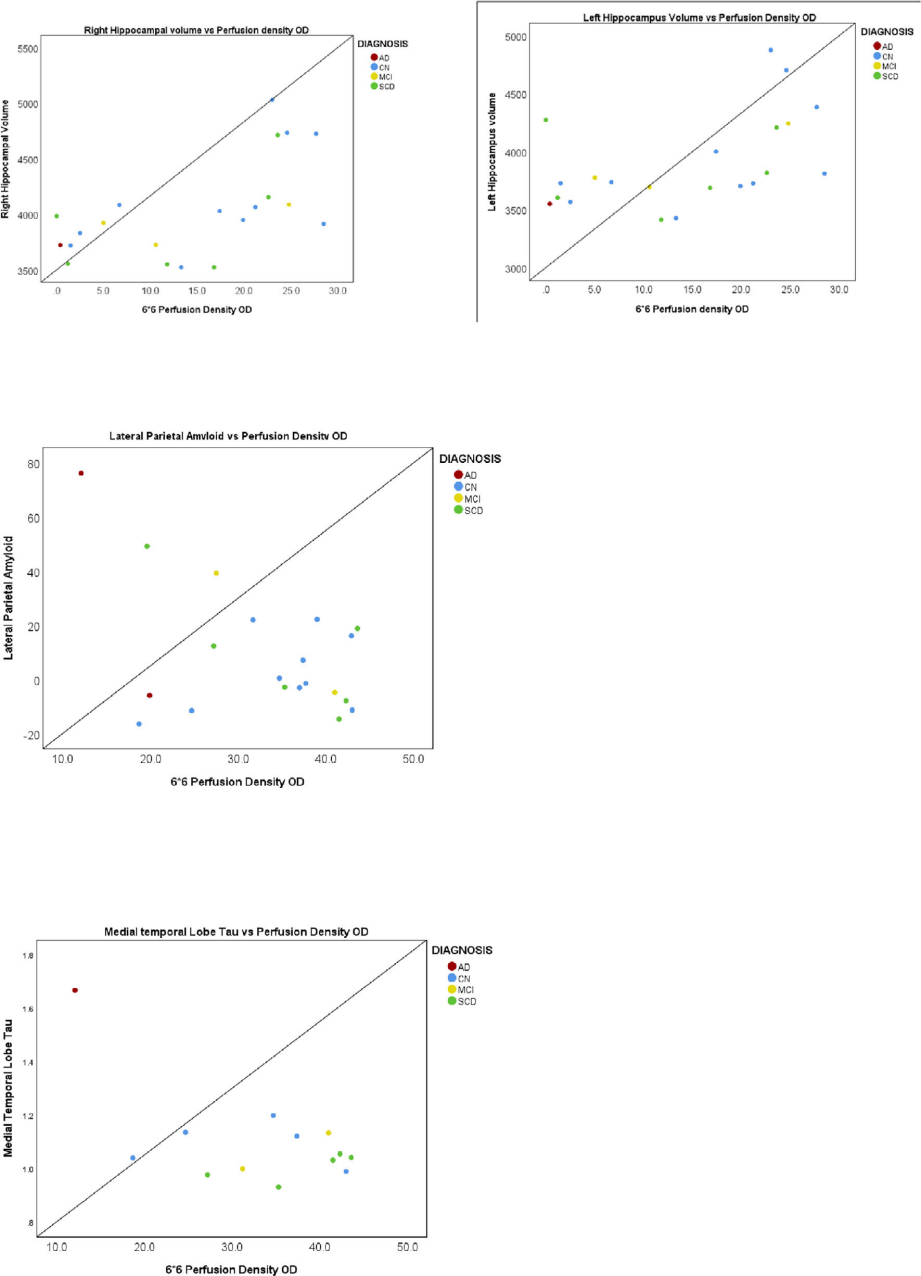# Relationship of retinal vasculature with measures of amyloid, tau, and neurodegeneration across the AD continuum

**DOI:** 10.1002/alz.092622

**Published:** 2025-01-09

**Authors:** Sunu Mathew, Devin Mackay, Eileen F. Tallman, Rachael Deardorff, Savannah Hottle, Aaron Vosmeier, David G. Clark, Martin R. Farlow, Jared R. Brosch, Sujuan Gao, Liana G. Apostolova, Andrew J. Saykin, Shannon L. Risacher

**Affiliations:** ^1^ Indiana University School of Medicine, Indianapolis, IN USA; ^2^ Department of Neurology, Indiana University School of Medicine, Indianapolis, IN USA; ^3^ Indiana Alzheimer's Disease Research Center, Indianapolis, IN USA; ^4^ Stark Neurosciences Research Institute, Indiana University School of Medicine, Indianapolis, IN USA; ^5^ Indiana Alzheimer's Disease Research Center, Indiana University School of Medicine, Indianapolis, IN USA; ^6^ Department of Radiology and Imaging Sciences, Indiana University School of Medicine, Indianapolis, IN USA; ^7^ Indiana University, Indianapolis, IN USA; ^8^ Regenstrief Institute, Inc, Indianapolis, IN USA; ^9^ Indiana University Center for Aging Research, Indianapolis, IN USA; ^10^ Department of Medical and Molecular Genetics, Indiana University School of Medicine, Indianapolis, IN USA; ^11^ Indiana University Network Science Institute, Bloomington, IN USA; ^12^ Center for Neuroimaging, Department of Radiology and Imaging Sciences, Indiana University School of Medicine, Indianapolis, IN USA; ^13^ Department of Radiology and Imaging Sciences, Center for Neuroimaging, School of Medicine, Indiana University, Indianapolis, IN USA; ^14^ Department of Medical and Molecular Genetics, School of Medicine, Indiana University, Indianapolis, IN USA

## Abstract

**Background:**

The eye often reflects changes seen in the brain in neurodegenerative diseases. This study sought to examine the relationship of retinal vasculature measured using optical coherence tomography angiography (OCTA) with temporal lobe neurodegeneration, and cerebral amyloid and tau deposition, in older adults along the Alzheimer’s disease (AD) continuum.

**Method:**

Participants included 13 cognitively normal subjects, 5 with subjective cognitive decline (SCD), 7 with cognitive impairment (mild cognitive impairment [MCI] and AD) from the Indiana Memory and Aging Study at the Indiana ADRC. Participants were excluded from the study if they had significant eye disease determined to interfere with OCTA, non‐AD dementia, or exclusion for MRI or PET. OCTA scans were obtained from each eye to measure retinal vessel density and perfusion density. MRI scans were processed using Freesurfer v6 to measure medial (MTL) and lateral temporal lobe (LTL) volumes. LTL SUVR values were extracted from [18F]flortaucipir PET scans. Finally, the association between retinal perfusion and vessel density with hippocampal volume, MTL tau, and lateral parietal amyloid was assessed using a partial Pearson correlation, covaried for age, sex, and diagnosis. p<0.05 was considered significant.

**Result:**

Retinal vessel and perfusion density were decreased in patients with AD. The right and left hippocampal volume were significantly correlated with retinal vessel density and perfusion density in the right eye and left hippocampal volume was correlated with vessel density in the left eye, but it did not reach significance. The retinal vessel density and perfusion density in the right eye correlated significantly with lateral parietal lobe amyloid and medial temporal lobe tau. Finally, the total gray matter volume correlated significantly with the retinal vessel density and perfusion density in the right eye and inversely with the foveal avascular zone in the right eye.

**Conclusion:**

Retinal perfusion and vessel density correlates with hippocampal atrophy, and general atrophy of the gray matter. It is also significantly correlated with the deposition of amyloid and tau in the brain. Imaging the retinal vasculature may represent a useful biomarker to screen patients at risk for AD prior to more invasive and prolonged testing.